# Visual Stimulus-Specific Adaptation in the Avian Imc and Its Effect on Target Decoding Performance

**DOI:** 10.3390/ani16172655

**Published:** 2026-08-24

**Authors:** Jiangtao Wang, Keli Yu, Ruohan Li, Liuyang Ma, Lipeng Zhang, Minjie Zhu, Zhizhong Wang

**Affiliations:** 1 School of Electrical and Information Engineering, Zhengzhou University, Zhengzhou 450001, China; jiangtaowang@zzu.edu.cn (J.W.); ykl@zzu.edu.cn (K.Y.); liruohan@stu.zzu.edu.cn (R.L.); zhanglipengdjj@zzu.edu.cn (L.Z.); zhumj@zzu.edu.cn (M.Z.); 2 The 27th Research Institute of China Electronics Technology Group Corporation, Zhengzhou 450047, China; mliuyang1119@163.com

**Keywords:** avian vision, Imc, stimulus-specific adaptation, local field potential, target decoding

## Abstract

SSA serves as a fundamental mechanism for optimizing sensory processing by selectively reducing neural responses to prevalent stimuli. While well-characterized in mammals, its evolutionary conservation and functional impact in avian species, particularly on neural coding and decoding, remain largely unexplored. Here, we investigated visual SSA in the avian nucleus Imc by simultaneously analyzing LFP spectral power and single-unit spiking activity in pigeons. During exposure to constant-order moving dot stimuli, repeated presentations induced sustained suppression of both low-gamma-band (31–70 Hz) and high-gamma (71–120 Hz) LFP power, which recovered upon novel stimulus introduction. A similar adaptive pattern was observed in spike firing rates. Stimulus-specific indices revealed that over 80% of Imc neurons exhibited SSA in gamma-band LFP energy, compared to 65.6% based on spike rates. To quantify the functional consequence, we employed machine learning decoders to assess the discrimination of moving targets. Prior to adaptation, decoding based on the spike rate achieved up to 81.8% accuracy, outperforming LFP-based decoding. Critically, SSA may impair the encoding of directional information in sustained responses, reducing the decoding accuracy of classifiers using spike rates, LFP features, or their combinations to near-baseline levels. These findings indicate that gamma-band LFP energy in the Imc primarily reflects stimulus salience, whereas spike rates concurrently encode both identity and salience. This study elucidates the differential roles SSA and its effect on multiplexed neural codes, advancing the understanding of invariant visual representation in the avian brain.

## Simple Summary

Stimulus-specific adaptation (SSA) is a neural mechanism that selectively reduces responses to repeated, predictable stimuli while preserving sensitivity to novel, unexpected ones, thereby optimizing sensory processing. Although extensively studied in mammals, SSA in the avian visual system remains poorly understood. This study investigated visual SSA in the nucleus isthmi pars magnocellularis (Imc), a key inhibitory structure in the pigeon midbrain attention network. We simultaneously recorded spike firing rates and local field potentials (LFPs) while presenting moving dot stimuli in alternating directions. Our results suggest that Imc neurons exhibit SSA: responses to repeated stimuli progressively declined but recovered upon direction change, with gamma-band LFP energy showing stronger adaptation than spike firing rates. Using machine learning decoders, we found that before adaptation, spike rates effectively encoded motion direction. However, SSA may impair the encoding of directional information across all signal types, reducing decoding accuracy to near chance levels. These findings indicate that gamma-band LFP primarily reflects stimulus salience, while spikes encode both identity and salience. This study advances our understanding of how evolutionarily conserved adaptation mechanisms balance sensitivity to novelty and suppression of redundancy in the avian brain.

## 1. Introduction

Neuronal adaptation is a fundamental mechanism that optimizes sensory processing by dynamically adjusting sensitivity to persistent inputs, thereby enabling the efficient allocation of limited metabolic and computational resources [1,2,3,4]. A refined form of this mechanism is SSA, characterized by a selective reduction in neural responses to frequent, predictable stimuli while maintaining responsiveness to rare or novel stimuli [1,5,6]. SSA was initially characterized in the mammalian auditory cortex and is now widely recognized as a neural correlate for novelty or change detection, serving as a foundational element for predictive coding theories [7,8,9]. SSA has been observed across various sensory modalities and throughout different levels of ascending sensory pathways in mammals, including the inferior colliculus, thalamic reticular nucleus, and somatosensory cortex [5,10,11,12,13]. The development of SSA during adolescence has also been explored, showing stability from early postnatal stages in the mouse lemniscal auditory system [14].

Despite extensive research on SSA in mammals, its evolutionary conservation and functional expression in the avian brain, which has undergone independent evolution for over 300 million years, remain significantly less explored. Investigating SSA in birds provides a unique opportunity to discern convergent computational principles from specialized visual systems shaped by distinct ecological niches and evolutionary pressures [15,16]. The avian midbrain tectofugal pathway, analogous to the mammalian retinotectal/collicular pathway, plays a dominant role in visual processing, especially for motion and spatial attention [17,18]. A crucial node within this pathway is the nucleus Imc, a GABAergic nucleus that generates stimulus salience [16,19]. The Imc receives topographic input from the optic tectum (OT), the avian homolog of the mammalian superior colliculus, and provides broad, inhibitory feedback [19,20]. This circuit implements a neural algorithm for spatial competition, effectively acting as a “salience map” [18]. Such a mechanism allows birds to rapidly select behaviorally relevant visual targets, such as predators or food items, from complex and cluttered backgrounds, a capability of paramount importance for survival in natural environments [16,21,22]. Recent research highlights the indispensable role of the Imc in spatial saliency mapping, where neurons responding to strong stimuli while inhibiting those with weak stimuli, irrespective of spatial distance or specific visual feature values [18]. This process enables the rapid detection of critical targets against complex backgrounds.

Despite comprehensive investigations into the mechanisms of spatial competition within the Imc, the temporal dimension of stimulus selection, particularly through SSA, is relatively unexplored in the avian visual system. However, previous work has demonstrated visual SSA to motion direction within the optic tectum [15,23]. Given the tight reciprocal connectivity between the optic tectum and the Imc [24], it is plausible that similar adaptive mechanisms operate within the Imc itself. Theoretically, temporal saliency mapping through SSA would complement spatial saliency computation [25], enabling animals to detect stimuli that deviate not only from their spatial surroundings but also from their temporal context [16]. This dual mechanism would provide a comprehensive solution for identifying biologically significant events in both spatial and temporal domains, a critical capability in dynamic visual environments [16,23].

The neural correlates of adaptation have traditionally been studied through changes in spike firing rates [26,27]. However, complementary information about population-level dynamics can be obtained from LFPs, which reflect the summed synaptic activity of neuronal ensembles within a region, typically spanning approximately 250–500 µm from the recording electrode [28]. LFPs are often decomposed into canonical frequency bands, with gamma-band oscillations particularly implicated in attention-related processes and feature binding [29,30]. Studies comparing decoding performance based on spikes versus LFPs have revealed interesting dissociations: while spike-based decoding generally outperforms LFP-based approaches for stimulus identity or motor intention, both signal types can achieve comparable accuracy for decoding attentional states [30]. Thus, spikes and LFPs may encode complementary aspects of sensory processing, with LFPs potentially reflecting more global brain states and spikes carrying more precise stimulus-specific information [31].

In the context of avian visual processing, the functional contributions of spike versus LFP signals remain poorly characterized, particularly regarding how adaptation modulates these different neural representations. This knowledge gap is significant because understanding the differential effects of SSA on multiple neural signals could reveal how adaptation optimizes information processing at both local circuit and population levels. Furthermore, while SSA is hypothesized to enhance the detection of novel stimuli by suppressing responses to predictable inputs, its functional consequences for downstream readout mechanisms, particularly the ability to discriminate between different object categories or motion directions, have not been systematically investigated in the avian brain [16,23].

To address these knowledge gaps, this study was designed to investigate visual SSA in the pigeon Imc employing a dual-modal approach. This involved simultaneously recording LFPs and single-unit activity during exposure to repetitive moving dot stimuli [16]. The aims of this study include: (1) establishing the existence of visual SSA in the Imc through both spike-rate attenuation and LFP spectral power suppression; (2) quantitatively comparing the prevalence and strength of SSA across neural response characteristics, hypothesizing that population-level LFP signals might exhibit distinct adaptive properties compared to single-unit spikes; and (3) evaluating the functional consequence of SSA by assessing its impact on the performance of machine learning decoders trained to discriminate object motion direction. By addressing these questions, this research would aim to advance the understanding of how evolutionarily conserved adaptive mechanisms operate in the avian visual system, particularly within a key node of the attention network. The findings will have implications for understanding the neural basis of behaviorally relevant visual processing in birds and could provide comparative insights into general principles of sensory adaptation across vertebrate lineages [16,23].

## 2. Materials and Methods

### 2.1. Ethical Approval and Animal Subjects

All experimental procedures were conducted in compliance with the Animals Act, 2006 (China), and received formal approval from the Life Science Ethical Review Committee of Zhengzhou University. Adult domestic pigeons (*Columba livia*) of both sexes (body weight range: 350–450 g) served as experimental subjects. The birds were maintained in individual wire mesh enclosures under standardized environmental conditions featuring a 12 h light–dark photocycle with unrestricted access to food and water. Given the documented absence of significant sex-based differences in subcortical neuronal responses within the relevant literature and consistent methodological approaches in SSA research [10,11,32,33,34], experimental groups were not segregated by sex.

### 2.2. Surgical Preparation and Electrophysiological Recording

The experimental protocols employed in this study were based on established methodologies [35]. Prior to surgery, pigeons were anesthetized via intraperitoneal injection of 20% urethane (dosage: 1 mL per 100 g of body weight). Deep anesthesia was confirmed by the absence of ocular, auditory, and foot-pinch withdrawal reflexes. Each subject was then secured in a stereotaxic apparatus (model ST-5ND-B; Chengdu Instrument, Chengdu, China). Throughout the procedure, the right eye was maintained open and hydrated with saline, while the left eye was occluded. A cranial window was created over the left dorsal brain to access the Imc. Following a dural incision made with a syringe needle, electrode penetrations into the Imc were performed. Core body temperature was regulated at normal temperature (approximately 41 °C) using a heating pad.

Neural activity was recorded using a 16-channel microelectrode array (4 × 4 configuration, Clunbury Scientific, Bloomfield Hills, MI, USA) advanced into the Imc with a micromanipulator. The Imc was targeted using a dorsoventral penetration angled 5 degrees medially to avoid major vasculature. Recordings were initiated approximately 30–40 min after urethane administration, and the recording sessions typically lasted 4–6 h while the depth of anesthesia was monitored throughout the experiment. Recording sites were identified based on stereotaxic coordinates and the characteristic physiological signatures of Imc neurons, which include high baseline firing rates and a distinctive vertically elongated receptive field structure—a unique feature within the avian midbrain. Final anatomical verification of Imc targeting was achieved post hoc using lesion techniques described previously [36].

Extracellular spike signals were sampled at 30 kHz and band-pass filtered (250–5000 Hz) for unit isolation. Concurrently, LFPs were amplified (4000×), low-pass filtered (0–250 Hz), and digitized at 2 kHz using a Cerebus^®^ acquisition system (Blackrock Microsystems, Inc., Salt Lake City, UT, USA). All subsequent analyses were performed offline with custom scripts in MATLAB (version R2022b, Natick, MA, USA).

### 2.3. Visual Stimulation Paradigms

Visual stimuli were generated and temporally controlled using the Psychophysics Toolbox (Psychtoolbox-3; www.psychtoolbox.org, accessed on 10 January 2019) operating within MATLAB on a Windows computing platform, with precise synchronization to electrophysiological recording. Stimuli were presented monocularly to the right eye using an LED monitor (Philips 558M1RY; monitor size: 1209.6 × 680.4 mm; 3840 × 2160 pixels) positioned 40 cm from the subject, subtending 112° vertically and 80° horizontally.

To map the receptive fields (RFs) of Imc neurons, visual stimuli consisted of a black dot (1.3° in diameter) presented against a gray background, which moved randomly at 30°/s along a series of parallel trajectories on a tangent LED monitor placed in front of the pigeon. Prior to formal experiments, the RFs were preliminarily estimated by manually moving a dot across the screen using a simple program while simultaneously observing the evoked firing responses.

**Figure 1:**
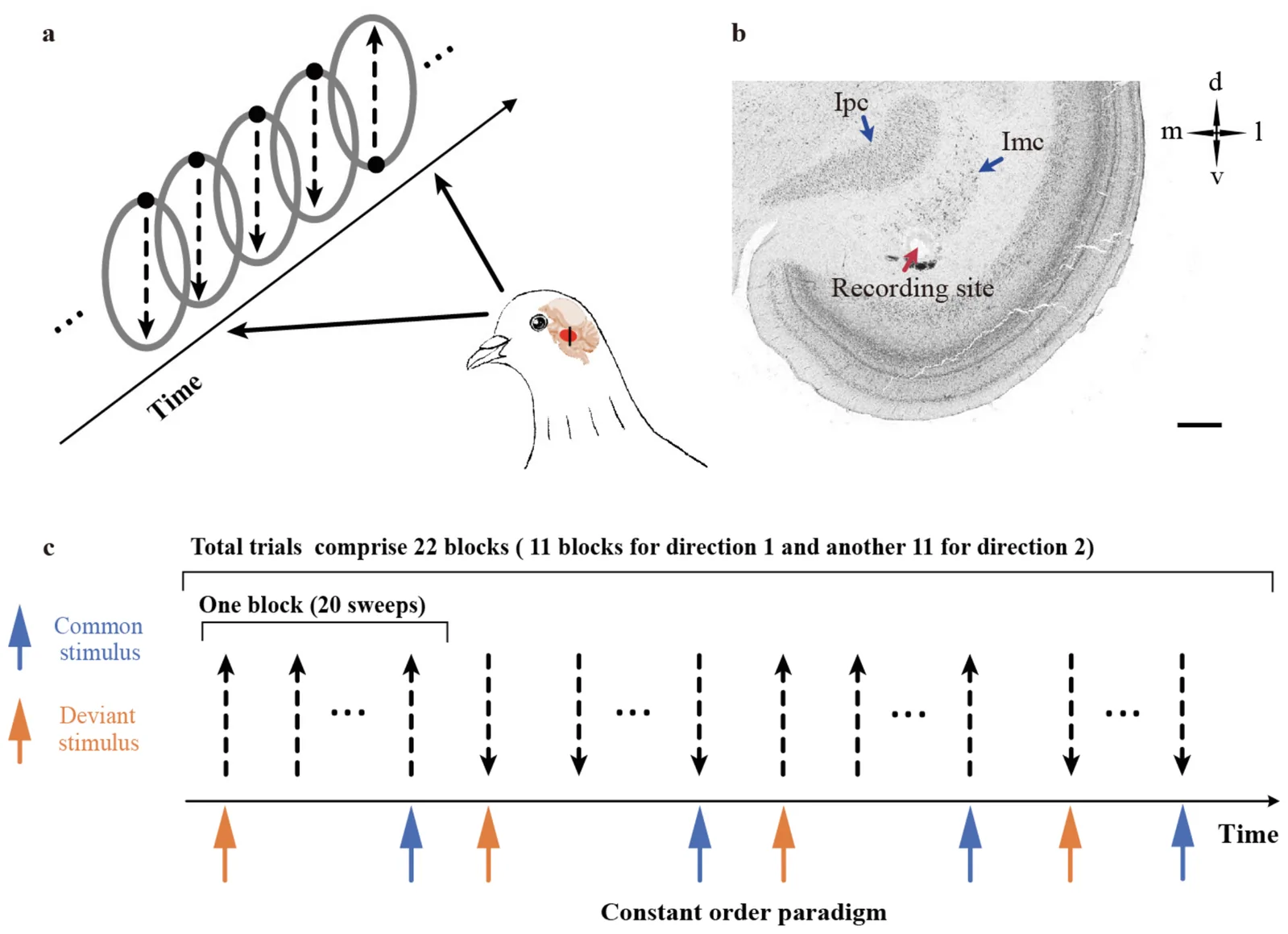
Experimental paradigm and histological identification: (**a**) schematic of the constant-order paradigm. Visual stimuli (a moving dot) traversed the receptive field (RF, gray ellipse) center of the recorded Imc unit in two directions: upward (direction 1) and downward (direction 2). The motion paths for both directions were spatially overlapped. The red ellipse in the brain schematic indicates the location of the Imc. The black vertical line denotes the coronal plane of the section shown in (**b**). (**b**) A Nissl-stained coronal section (40 µm) used for anatomical verification of the recording site. The blue arrow points to the Imc, and the red arrow indicates the estimated location of the recorded unit. Scale bar: 1 mm. Abbreviations: d, dorsal; v, ventral; m, medial; l, lateral. (**c**) Detailed trial structure of the constant-order paradigm for probing stimulus-specific adaptation. The paradigm consisted of 22 blocks, with direction 1 and direction 2 blocks alternating. Each block contained 20 stimulus sweeps of the same direction. Within a block, the first sweep was designated as the deviant stimulus (orange arrow) and the last sweep as the common stimulus (blue arrow).

As Figure 1c shows, to investigate stimulus-specific adaptation, a sequential paradigm called constant order [6,37] was implemented: Stimuli consisted of a black circular target (diameter: 1.3°) against a uniform gray background (luminance: 118 cd/m^2^; target luminance: 0.2 cd/m^2^), as Figure 1a shows. The target initially positioned extraneous to the Imc RF executed 20 consecutive movements in one direction, followed immediately by 20 movements in the antipodal direction along identical trajectories. The path of upward and downward movement of the target completely overlaps and passes through the center of the recorded Imc unit’s receptive field. Each unidirectional sequence constituted a “block”, with eleven alternating blocks comprising the complete paradigm (total trials: 440). Experimental variables included target velocity (30°/s) and inter-stimulus interval (ISI: 1 s). Within analytical frameworks, the initial directional presentation per block was designated the “deviant stimulus”, while the terminal (20th) presentation served as the “common stimulus”.

### 2.4. Data Processing and Analysis

For the raw data acquired, LFP signals within the 1–250 Hz range were first extracted using a Butterworth filter. Subsequently, a dedicated notch filter was applied to remove 50 Hz of powerline interference. The processed signals were then band-pass filtered into six canonical frequency bands for analysis: delta (1–4 Hz), theta (5–8 Hz), alpha (9–12 Hz), beta (13–30 Hz), slow gamma (31–70 Hz), and high gamma (71–120 Hz). Spike firing rates and LFP spectral powers were calculated within a temporal window spanning from 200 ms post-stimulus onset to its offset, and for SSA quantification, statistical analyses included repeated-measures ANOVA, linear trend tests, and *t*-tests (α = 0.05).

**Figure 2:**
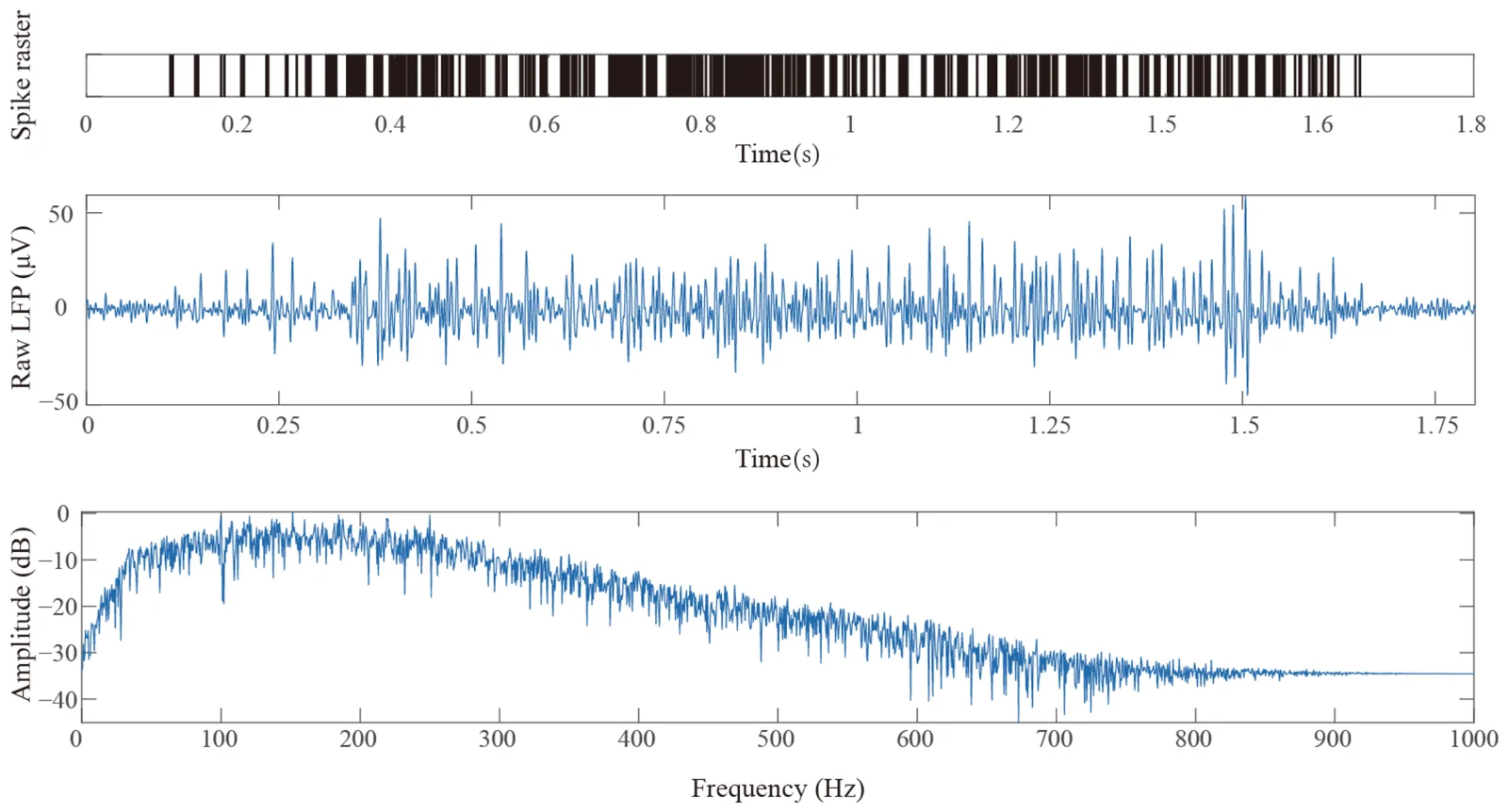
Representative neural activity patterns to the first sweep from a sample single Imc neuron. (**Top panel**) Spike raster plot displaying the temporal distribution of action potentials over a recording epoch. Each vertical tick represents an individual spike, illustrating the firing pattern of the isolated neuron (sorted with the Wave_clus toolbox). (**Middle panel**) Simultaneously recorded raw LFP trace from the same site over the identical time window, reflecting the summed synaptic and population activity of the local circuit. (**Bottom panel**) Amplitude spectrum of the LFP signal (0–1000 Hz), plotted in decibels (dB). The spectrum reveals the frequency composition of the oscillatory activity, with prominent power concentrated in the gamma-frequency range.

Single-unit isolation was performed using the “Wave_clus” toolbox [38], an unsupervised algorithm for efficient spike detection and sorting. Units were retained for further analysis only if they exhibited an inter-spike interval violation rate of less than 5% within a 1.5 ms refractory period [36], as the top panel in Figure 2 shows.

Receptive fields were assessed by computing the firing rate of each single unit across the screen. In SSA research, the adaptation effects of interest are primarily manifested in the modulation of sustained responses rather than the onset transient, thus the evoked response window was set 200 ms post-stimulus onset to its offset. Although our analysis revealed some degree of RF overlap among simultaneously recorded neurons, to avoid potential cross-interference during visual stimulation, we selected only those neurons with non-overlapping RFs for subsequent constant-order visual stimulation.

The amplitude–frequency characteristics (i.e., power spectral density) of the preprocessed LFP signals were obtained through Fast Fourier Transform (FFT) using a Hanning window for spectral leakage reduction, with the resultant spectra used to quantify band-limited power. Receptive fields (RFs) of Imc units were mapped by computing their firing rates across screen coordinates. Final response maps were generated by averaging across all trials, and regions where the evoked activity significantly exceeded the baseline were defined as the unit’s RF.

To mitigate onset effects in the visual SSA analysis, the initial two blocks of each recording were excluded. Within each subsequent block, the response to the first stimulus presentation was designated as the deviant response, while the response to the final presentation of the same stimulus served as the common (adapted) response.

The strength of SSA for each motion direction was quantified using a standardized stimulus index (SI) [6,37,39], calculated as follows:

$$SI_{i}=\frac{R_{i}d-R_{i}c}{R_{i}d+R_{i}c}$$

where $R_{i}d$ and $R_{i}c$ represent the mean neural responses to the deviant and common stimuli for a given direction, respectively, and *i* represents the motion direction. Indices for the two tested directions are abbreviated as *SI*_1_ and *SI*_2_. A fundamental property of stimulus-specific adaptation is that the sum of *SI*_1_ and *SI*_2_ is expected to be positive, whereas non-specific adaptation would yield a sum near zero. Therefore, units with a positive sum (*SI*_1_ + *SI*_2_ > 0) were classified as exhibiting SSA, consistent with established population-level criteria [40,41]. Units demonstrating positive SI values for both directions (*SI*_1_ > 0 and *SI*_2_ > 0) indicated a robust preference for the initial, novel stimulus across conditions [42]. For population-level analysis, responses at each recording site were normalized to their maximum before averaging across the population.

### 2.5. Histological Verification

Following electrophysiological recordings, precise anatomical localization of recording sites was achieved through electrolytic lesioning (0.2 mA direct current, 30 s duration via recording electrode). Subjects were subsequently euthanized via urethane overdose and underwent transcranial perfusion with phosphate-buffered saline (PBS) followed by 4% paraformaldehyde (PFA) in PBS. Extracted brains underwent post-fixation (4% PFA, 12 h), cryoprotection (30% sucrose, 4 °C, 24 h), and cryosectioning (40 µm coronal sections). Tissue sections were Nissl-stained with Cresyl violet and microscopically examined to confirm electrode placements within the Imc nucleus (Figure 1b). Only data from histologically verified Imc units were incorporated in subsequent analyses.

### 2.6. Decoding

To investigate the effect of adaptation on the encoding capability of Imc neurons for moving targets, the first trial of each sequence was defined as the pre-adaptation target for direction 1 and the 420th trial as the post-adaptation target for direction 1. Similarly, the 21st trial was defined as the pre-adaptation target for direction 2 and the 440th trial as the post-adaptation target for direction 2. The sets of neural response features (temporal window spanning from 200 ms post-stimulus onset to its offset) evoked by the two motion directions were used as inputs to the decoding models. In this study, seven feature sets were adopted: spike firing rates, low-gamma (LG) energy, high-gamma (HG) energy, their pairwise combinations, and the triple combination. Each feature was calculated within a temporal window spanning from 200 ms after stimulus onset to stimulus offset.

Three decoders were used for target decoding: support vector machine (SVM), linear discriminant analysis (LDA), and k-nearest neighbors (KNN). Eighty percent of the total response data served as the training set to train the decoding models, and the remaining 20% served as the test set for validating model performance. The baseline level of decoding accuracy was 50%.

## 3. Results

Fifteen pigeons were used in our study, and the neuronal signals were collected from fifty-five Imc neurons during the experiments; spike firing rates and LFPs were then extracted for all remaining analyses in this study.

### 3.1. Gamma-Band LFP Dominates Motion Stimulus Encoding in the Imc

LFP oscillations across specific frequency bands, including delta (1–4 Hz), theta (5–8 Hz), alpha (9–12 Hz), beta (13–30 Hz), and gamma (slow gamma: 31–70 Hz; high gamma: 71–120 Hz), are known to be involved in visual processing and cognitive functions. To determine which frequency bands in the Imc carry information about moving visual stimuli, we performed a spectral analysis of LFP signals. For a representative Imc neuron, the amplitude–frequency analysis revealed a distinct spectral profile where the power was markedly concentrated within the gamma range (Figure 2 middle and bottom panels).

To quantify this observation, the LFP signal from this neuron was decomposed into the standard frequency bands. The distribution of band-limited power was highly asymmetric, with the gamma bands accounting for most of the signal energy. The calculated energy proportions were as follows: delta (0.0001%), theta (0.0001%), alpha (0.0004%), beta (0.0662%), slow gamma (3.9526%), and high gamma (21.9471%).

We next assessed whether the above energy distribution pattern was a general property across the recorded neural population. Statistical analysis across 55 Imc neurons confirmed the consistent prevalence of gamma oscillations. The mean energy proportions for the population were: delta (0.0018%), theta (0.0042%), alpha (0.0073%), beta (0.2288%), slow gamma (7.3441%), and high gamma (19.9011%). The combined contribution of slow- and high-gamma bands was significantly greater than that of all lower frequency bands combined, and these results also echo previous research on other nuclei in the avian midbrain network [43,44]. Given the predominant role of gamma-band LFP, all subsequent analyses focused on the slow- and high-gamma-frequency components.

### 3.2. Stimulus-Specific Adaptation in Single Imc Neuron

**Figure 3:**
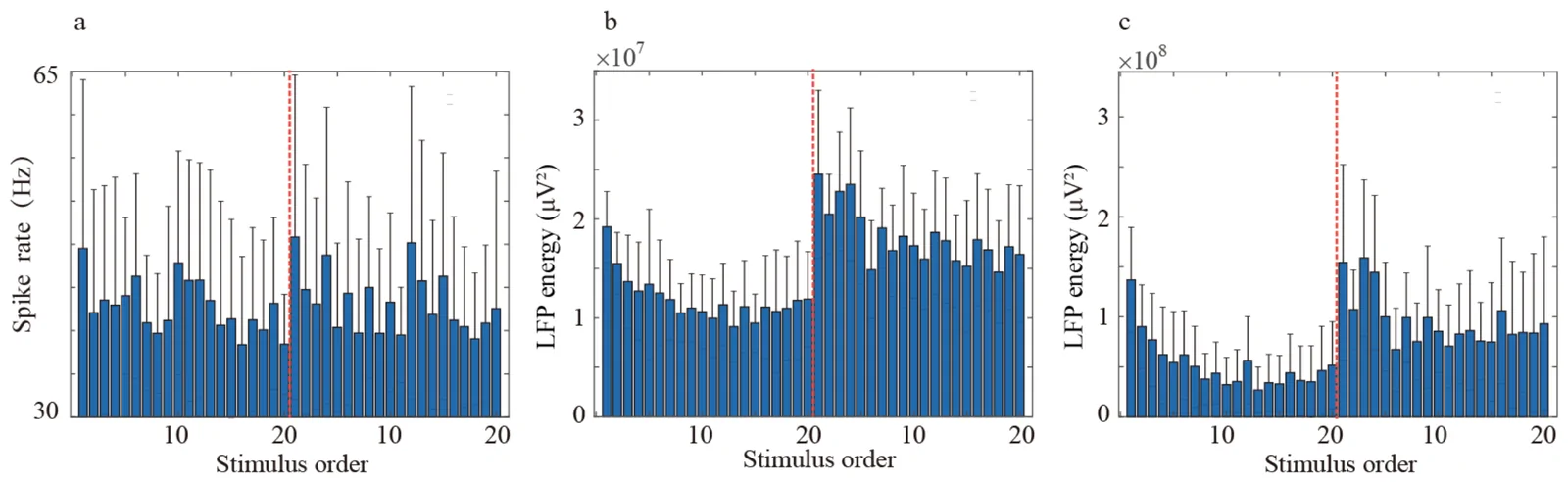
Neural response dynamics of a single neuron during constant-order stimulus presentation: (**a**) spike firing rate plotted against stimulus order within the sequence. Each motion direction (direction 1 and direction 2) consisted of 20 repeated presentations within each block, and the spike rates with the same order were averaged among blocks. A decreasing trend in firing rate with repetition was observed for both directions. The response to the first stimulus in a block (deviant) significantly exceeded that of the last one (common; One-way ANOVA, *p* < 0.05), with a clear recovery of response magnitude following the directional switch (One-way ANOVA, *p* < 0.05). (**b**) Mean power in the slow-gamma-frequency band (31–70 Hz) across the stimulus sequence. The power exhibited a significant decline with stimulus repetition (Kendall’s test: direction 1, τ = −0.4316, *p* < 0.01; direction 2, τ = −0.5263, *p* < 0.001). Similar to spiking activity, the response to deviant stimuli was significantly stronger than to common stimuli (One-way ANOVA, *p* < 0.05) and recovered upon direction change (One-way ANOVA, *p* < 0.05). (**c**) Mean power in the high-gamma band (71–120 Hz). The response also showed a significant decreasing trend with repetition (Kendall’s test: direction 1, τ = −0.4632, *p* < 0.005; direction 2, τ = −0.3158, *p* = 0.05), with deviant responses being significantly stronger than common responses (One-way ANOVA, *p* < 0.05) and rebounding after the direction switch (One-way ANOVA, *p* < 0.005). Error bars indicate SD, and the red dashed line was used to separate the two directions.

Building on established findings that adaptation primarily modulates neuronal spike firing rates, we first analyzed the response dynamics of a representative Imc neuron under the constant-order paradigm. As shown in Figure 3a, the spike response strength of the neuron was significantly suppressed during repetitive presentations of a motion direction. This suppression was reversible; the response recovered when the motion direction switched (One-way ANOVA, *p* < 0.05). Following this switch, the response strength again exhibited a decreasing trend with subsequent repetitions of the new direction. This pattern of suppression and recovery indicates the presence of SSA at the level of spiking activity.

We next asked whether SSA is also reflected in LFP signals. For the same neuron, we computed the energy in the slow-gamma and high-gamma bands. Statistical analysis revealed a pattern analogous to that observed for spike rates. The slow-gamma-band energy showed a significant decrease with stimulus repetition (Kendall’s test: direction 1: τ = −0.4316, *p* < 0.01; direction 2: τ = −0.5263, *p* < 0.001), as shown in Figure 3b. Consequently, responses to standard (repeated) stimuli were significantly attenuated compared to novel stimuli (One-way ANOVA, *p* < 0.05 for both directions) but recovered upon motion direction reversal (One-way ANOVA, *p* < 0.0005). Similarly, as Figure 3c shows, high-gamma-band energy followed a declining trend with repetition (Kendall’s test: direction 1: τ = −0.4632, *p* < 0.005; direction 2:τ = −0.3158, *p* = 0.05), with responses to novel stimuli being significantly stronger than to standard ones (One-way ANOVA, *p* < 0.05 for both directions). The neuron maintained sensitivity to novel stimuli despite adaptation (One-way ANOVA, *p* < 0.005).

Collectively, these results demonstrate that Imc neurons exhibit an adaptive property characterized by response suppression to repeated stimuli and preserved sensitivity to novel stimuli. To further quantify this adaptation, the stimulus-specific adaptation index (SI) was calculated for the example neuron. For spike firing rates, the SI values were 0.4069 for direction 1 and 0.2350 for direction 2, confirming the presence of visual SSA. For slow-gamma responses, the SI values were 0.2352 and 0.1978 for directions 1 and 2, respectively; the corresponding high-gamma SI values were 0.4534 and 0.2483. These results indicate that the power changes in the slow- and high-gamma frequency bands are consistent with SSA, and that the effect of SSA on Imc neural responses is reflected not only in the spike firing rates but also in the LFP band-limited energy.

### 3.3. SSA on the Population Neuron Level

**Figure 4:**
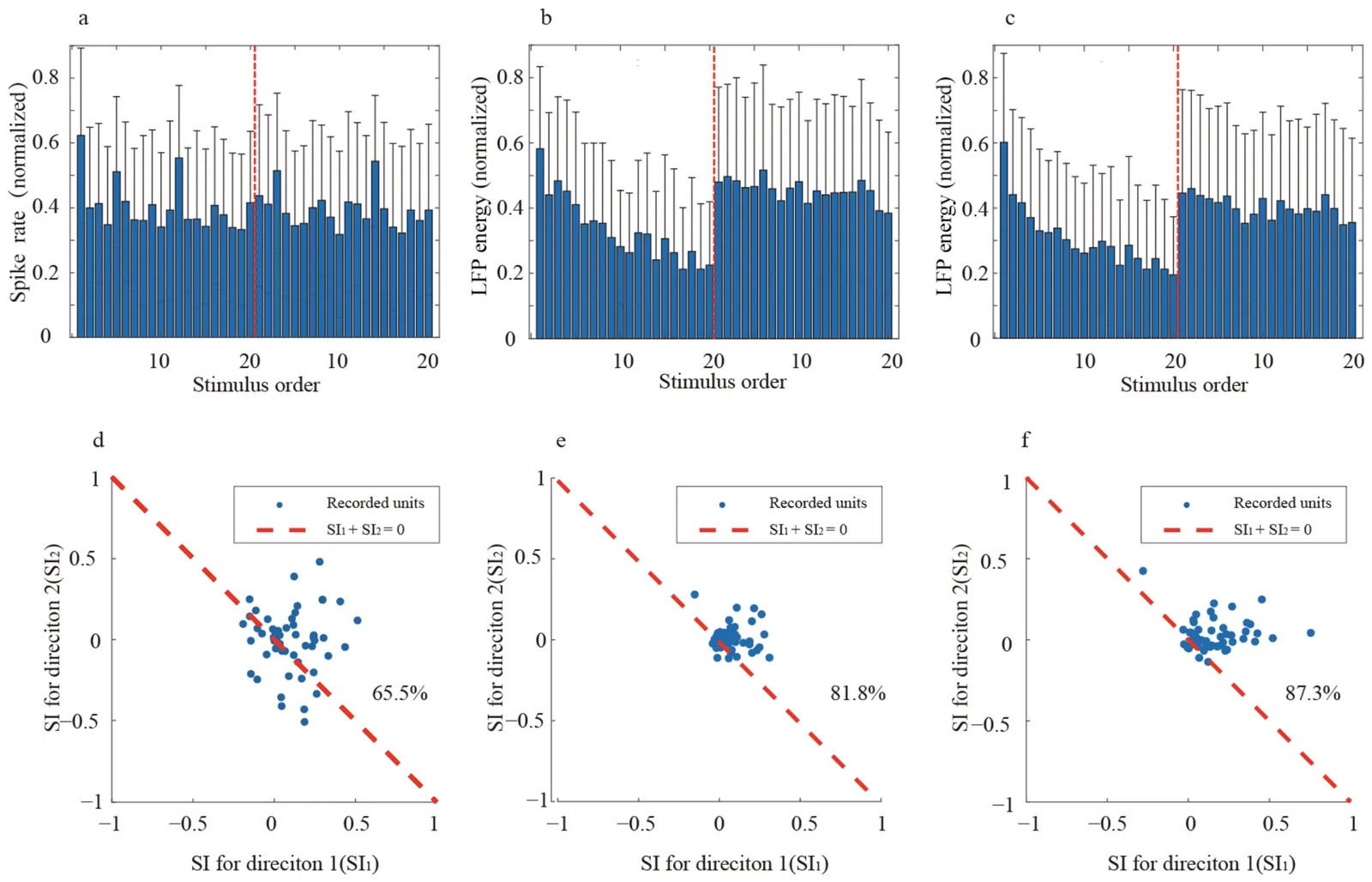
Population-level dynamics and stimulus selectivity of SSA in the Imc: (**a**) population-averaged, normalized spike firing rates across repeated stimulus presentations for two motion directions. A decreasing trend with repetition was observed. Response magnitude recovered following a directional switch (One-way ANOVA, *p* < 0.05). (**b**) Normalized slow-gamma-band (31–70 Hz) LFP power averaged across the population. Power showed a significant decrease with stimulus repetition (Kendall’s test: direction 1, τ = −0.5684, *p* < 0.0005) and recovered upon direction change (One-way ANOVA, *p* < 0.001). (**c**) Normalized high-gamma-band (71–120 Hz) LFP power. A similar adaptive pattern was observed (Kendall’s test: direction 1, τ = −0.5895, *p* < 0.0005), with sensitivity to novel stimuli preserved. (**d**) Distribution of the stimulus-specific adaptation index (SI) based on spike rate for individual neurons: 65.5% of neurons (36/55) exhibited SSA (SI > 0). (**e**) Distribution of SI based on slow-gamma LFP power: 81.8% of sites (45/55) showed SSA. The population SI was 0.3729 for direction 1 and 0.005 for direction 2. (**f**) Distribution of SI based on high-gamma LFP power: 87.3% of sites (48/55) showed SSA, with population SI values of 0.4609 (direction 1) and 0.0295 (direction 2). In (**d**–**f**), the red dashed line indicates the boundary SI_1_ + SI_2_ = 0. Error bars indicate SD, and the red dashed line was used to separate the two directions.

To examine SSA dynamics at the population level, normalized responses were averaged across all recording sites. As Figure 4a shows, the average spike firing rate of Imc neurons was suppressed after repetitive stimulus presentations. This suppression was reversible, with the population response recovering upon motion direction switch, after which it again decreased with subsequent repetitions of the new direction.

We next analyzed population-level SSA in gamma-band LFP oscillations. The averaged slow-gamma-band energy exhibited progressive attenuation with stimulus repetition (Kendall’s test: direction 1: τ = −0.5684, *p* < 0.0005). Responses to standard stimuli were significantly weaker than to novel stimuli (One-way ANOVA, *p* < 0.05 for both directions), with robust recovery to novelty following adaptation (One-way ANOVA, *p* < 0.001; Figure 4b). A similar adaptive pattern was observed in the high-gamma band (direction 1: τ = −0.5895, *p* < 0.0005), while sensitivity to novel stimuli was preserved (One-way ANOVA, *p* < 0.001), as shown in Figure 4c.

The strength of this adaptation was quantified using the SI. Population SSA was pronounced for direction 1, with SI values of 0.3729 for the slow-gamma band and 0.4609 for the high-gamma band. The effect for direction 2 was more modest, with corresponding SI values of 0.0050 and 0.0295, respectively.

Finally, we determined the proportion of recording sites exhibiting SSA (SI > 0). Based on spike firing rates, 65.5% of Imc neurons showed visual SSA (Figure 4d). This prevalence was higher for LFP measures: 81.8% of sites exhibited SSA in the slow-gamma band (Figure 4e) and 87.3% in the high-gamma band (Figure 4f). This indicates that SSA is a widespread phenomenon in the Imc and is more consistently detected in the spectral features of population LFP signals than in single-unit spike rates.

### 3.4. Decoding Moving Targets Based on Spike and LFP Features

In neuroscience, decoding analysis serves to identify neural response properties that encode specific stimuli or cognitive states. By examining features extracted from spike trains and LFPs, we can assess the dynamic changes in neural representations during target-related information processing. Furthermore, decoding allows for the evaluation of which feature sets most effectively carry information about the target. In this study, to quantify motion direction discriminability from Imc activity before and after adaptation, target information was decoded using three distinct classifiers, SVM, LDA, and KNN, which were trained to distinguish two motion directions using seven feature sets: spike firing rates, low-gamma (LG) energy of LFPs, high-gamma (HG) energy of LFPs, and their pairwise and triple combinations.

**Figure 5:**
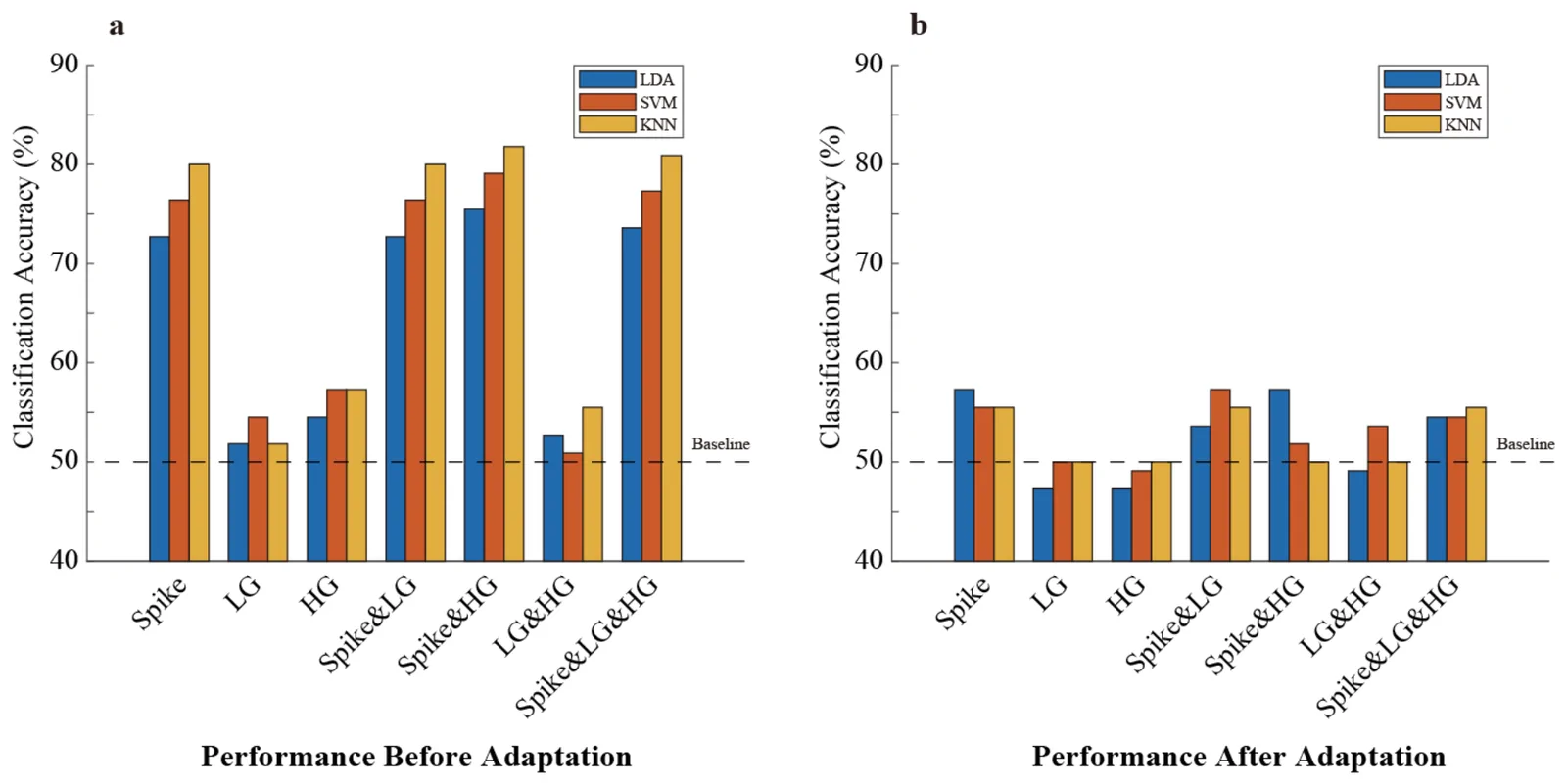
Target decoding performance comparison using different neural response features and machine learning algorithms. (**a**) Classification accuracy before adaptation across seven distinct feature combinations and three machine learning algorithms. The feature combinations systematically evaluate individual and integrated neural signatures: spike rates, low-gamma energy (LG) of LFPs, high-gamma energy (HG) of LFPs, and their pairwise and triple combinations. Three classification algorithms are employed: LDA (blue bars), SVM (brown bars), and KNN (yellow bars). The KNN algorithm achieves superior performance, particularly with Spike&HG (81.8%), Spike&LG&HG (80.9%), and Spike&LG (80.0%) combinations. SVM exhibits competitive performance with peak accuracy of 79.1% using Spike&HG features, while LDA shows moderate performance with maximum accuracy of 75.5% for the same feature combination. Notably, individual features show varying baseline performances: Spike features achieve 72.7–80.0% accuracy, LG features range from 51.8 to 54.5%, and HG features vary between 54.5 to 57.3% across algorithms. (**b**) Classification accuracy after adaptation reveals substantial performance degradation across all feature combinations and algorithms, indicating the profound impact of neural adaptation on decoding reliability. The performance reduction is algorithm-dependent: KNN shows the most significant decline (average decrease of 17.3 percentage points), with post-adaptation accuracies converging to 50.0–55.5% across most feature combinations. SVM demonstrates moderate resilience (14.3 percentage point average decrease), maintaining relatively better performance with Spike&LG (57.3%) and LG&HG (53.6%) combinations. LDA exhibits the most robust performance (12.4 percentage point average decrease), with Spike features showing the best post-adaptation accuracy (57.3%). The convergence of post-adaptation accuracies is toward chance level (50%).

Prior to adaptation, spike-rate features substantially outperformed LFP-based approaches (Figure 5a). KNN achieved the highest accuracy using spike features alone (80.5%), followed by SVM (77.0%) and LDA (73.0%), highlighting the primary role of spike timing in encoding stimulus identity within the Imc. In contrast, decoding based solely on low-gamma energy remained near chance levels (51.5–56.0%), while high-gamma energy performed slightly better (54.0–58.0%), indicating that gamma-band oscillations alone carry limited directional information.

Feature combinations significantly enhanced decoding performance. The spike and HG combination yielded the highest overall accuracy (KNN: 82.0%; SVM: 79.0%; LDA: 76.0%), representing a synergistic improvement over spike features alone. The spike and LG combination achieved comparable performance (KNN: 81.0%; SVM: 78.0%; LDA: 74.0%), as did the triple combination spike and LG and HG (KNN: 81.0%; SVM: 78.0%; LDA: 53.0%). These results demonstrate that spike timing and gamma-band oscillations encode complementary aspects of motion direction information.

However, the addition of low-gamma energy to spike features provided no significant benefit over spike features alone, with spike and LG performance (KNN: 81.0%; SVM: 78.0%; LDA: 74.0%) comparable to spike-only decoding. Similarly, combining low- and high-gamma energies without spike information (LG and HG: KNN: 53.0%; SVM: 78.0%; LDA: 53.0%) failed to achieve above-baseline accuracy, confirming that gamma oscillations alone cannot effectively encode motion direction.

Following adaptation, decoding accuracy across all feature sets and classifiers was compromised, approaching baseline levels (55.0–59.0%; Figure 5b). Spike-rate features, previously the most informative, dropped to 50.0–56.0%. LFP-only features declined to 46.0–51.0%, effectively at chance. Critically, the pre-adaptation advantage of combined features was eliminated. The previously best-performing spike and HG combination collapsed to 52.0–56.0%, indistinguishable from spike-rate performance. The triple combination spike and LG and HG showed similar degradation (50.0–52.0%).

Algorithm-dependent differences in adaptation susceptibility were evident. KNN, which excelled before adaptation, exhibited the most substantial decline (average decrease of 25–30 percentage points), while LDA demonstrated relatively better resilience, maintaining 57.0% accuracy with spike and LG features.

## 4. Discussion

This study provides comprehensive evidence for SSA in the avian nucleus Imc, demonstrating its differential manifestations in spike-rate and LFP signals and its profound impact on object decoding performance. Our findings reveal that gamma-band oscillations in the LFP are particularly sensitive to repetitive stimulation, with over 80% of Imc recording sites exhibiting SSA in these frequency ranges. This exceeds the proportion of sites showing SSA in spike firing rates (65.6%), suggesting that population-level signals may be more susceptible to adaptation than single-unit activity in this brain region. Critically, we show that SSA dramatically reduces the performance of machine learning decoders in distinguishing targets moving in different directions, effectively returning decoding accuracy to near-baseline levels across all neural feature types. These results collectively support our hypotheses while revealing unanticipated dissociations between how spike and LFP signals encode stimulus properties and respond to adaptation.

### 4.1. SSA in the Avian Midbrain Attention Network

It is critical to distinguish between non-specific adaptation and stimulus-specific adaptation (SSA). Non-specific adaptation would predict a generalized reduction in neural responsiveness regardless of stimulus identity, which is incompatible with our observation that Imc neurons selectively reduce responses to repeated motion directions while maintaining sensitivity to novel directions (Figure 3 and Figure 4). The stimulus-selective nature of the observed adaptation strongly suggests that different motion directions are processed through distinct synaptic input pathways converging onto the same Imc neurons and that SSA reflects local, input-specific synaptic depression or receptor desensitization rather than a global change in neuronal excitability. The discovery of robust visual SSA in the Imc extends previous findings of adaptive phenomena in the avian optic tectum and aligns with the proposed role of the midbrain attention network in temporal saliency mapping [16]. The Imc’s position as a GABAergic inhibitory hub within this network suggests that SSA in this structure may serve to dynamically recalibrate competition between visual stimuli based on their recent occurrence history. When a stimulus becomes predictable through repetition, attenuated Imc responses could reduce inhibitory suppression of tectal neurons representing that stimulus, potentially freeing computational resources for processing novel inputs. This mechanism would implement a form of temporal contrast enhancement that complements the spatial contrast computations already established for the Imc. Recent research in avian species, such as pigeons and zebra finches, has identified precise layer structures and comprehensive layer-specific signatures in the optic tectum, offering a detailed molecular landscape for understanding visual processing [20]. This work further emphasizes the significance of adaptive mechanisms within the avian midbrain.

The particularly strong adaptation observed in gamma-band LFP power is noteworthy given the association between gamma oscillations and attention-related processes across species. Gamma oscillations are thought to facilitate coordinated activity among neuronal populations processing related features, and their suppression during stimulus repetition may reflect a reduction in the coordinated assembly of neurons representing predictable stimuli [16,29]. The subsequent restoration of gamma power upon novel stimulus presentation suggests that these oscillatory dynamics are rapidly reconfigured to process unexpected inputs, potentially enhancing their salience in downstream regions. This pattern aligns with observations in mammalian systems where gamma oscillations are modulated by attention and stimulus predictability [9,30], though direct comparisons between avian and mammalian systems must consider their divergent evolutionary trajectories and distinct visual system architectures [21]. For instance, studies in macaque V1 have shown that both repetition-related reductions in the firing rate and increases in gamma are specific to the repeated stimulus, with these effects showing persistence over minutes [29].

In this constant-order paradigm, upward (direction 1) and downward (direction 2) motions were employed to examine the presence of visual SSA. As depicted in Figure 4, the population-level visual SSA was more pronounced for direction 1 than for direction 2. The reduced SSA effect observed for downward motion may arise from two possibilities. One is that Imc neurons exhibit a diminished capacity for response adaptation to downward motion. The other is that adaptation does occur, but in a non-stimulus-specific manner. If the latter holds true, the neural response to a downward deviant stimulus should not significantly deviate from preceding responses, and vice versa. Consequently, a weaker SSA effect for direction 2 suggests that Imc neurons adapt less readily to downward-moving objects, thereby revealing a significant direction dependence of visual SSA. This directional bias in adaptation magnitude may hold ecological relevance for prey survival, as it may continuously maintain the animal’s vigilance against predators diving from higher visual space.

### 4.2. Differential Encoding in Spike and LFP Signals

Under the constant-order paradigm, the decoding performance based on average neural response features (firing rates and LFP energy) significantly decreased after adaptation, suggesting that SSA may impair the encoding of directional information in sustained responses. Therefore, incorporating analyses controlling for overall response magnitude and including response normalization or the temporal structure of neural responses would further enhance the robustness of decoding, and incorporating these features into the decoding framework represents an important direction for future research.

The dissociation between spike-based and LFP-based decoding performance before adaptation provides important insights into the complementary information carried by these signals [16]. The superior object discrimination achieved using spike rates (81.8% accuracy) compared to LFP features (near baseline) suggests that stimulus identity is more precisely represented in the timing of action potentials than in population-level oscillations within the Imc. This finding is consistent with studies in mammalian systems demonstrating that spike-based decoding generally outperforms LFP-based approaches for stimulus discrimination tasks [30].

Conversely, the greater prevalence and strength of SSA in LFP gamma power compared to spike rates suggests that population-level dynamics may be more sensitive to stimulus history. This differential sensitivity could reflect distinct underlying mechanisms: while spike-rate adaptation may primarily involve intrinsic cellular properties or synaptic depression at specific inputs [26], LFP adaptation might reflect broader network-level adjustments in rhythmic activity patterns and synchronization [9,28]. The finding that both signals show recovery upon novel stimulus presentation indicates that these adaptive processes are stimulus-specific rather than generalized fatigue, consistent with the defining characteristics of SSA [16]. This is further supported by models of efficient coding, which explain how neural response homeostasis and SSA arise from adjusting neuronal responsiveness to stimulus prevalence [2].

The combined results support a model in which spike timing in Imc neurons encodes both stimulus features and salience, while gamma oscillations primarily reflect salience-related processing. This division of labor would allow the Imc to simultaneously represent what is in the environment and which elements deserve priority in attention, with both representations being dynamically modulated by recent experience through SSA. The role of the Imc in the avian midbrain network for encoding stimulus salience and selection is further detailed by studies on the dynamics of stimulus selection, including reciprocal inhibitory projections among Imc neurons.

### 4.3. Functional Implications of SSA for Visual Processing

The dramatic reduction in decoding accuracy following adaptation has important implications for understanding how prior exposure shapes visual processing in the avian brain. By reducing the discriminability of neural representations for repeated stimuli, SSA may effectively “downgrade” their priority in attention and memory systems, allowing novel stimuli to dominate behavior. This mechanism would be ecologically advantageous for detecting potential threats or resources that deviate from recent experience [21]. For instance, detection of looming stimuli, crucial for survival, benefits from such selective processing [22].

The near-complete loss of decoding information across all feature types after adaptation suggests that SSA in the Imc induces a comprehensive reformatting of neural representations rather than merely scaling response magnitudes. This reformatting may involve changes in both the rate codes (firing frequencies) and temporal codes (precise spike timing and oscillatory synchronization) that collectively define stimulus representations [16,26]. Such comprehensive representational changes would make adapted stimuli difficult to distinguish not only for artificial decoders but potentially for downstream brain regions that read out Imc signals to guide behavior.

From a systems neuroscience perspective, these findings highlight how adaptation at an early stage of sensory processing (the midbrain attention network) can propagate to influence higher-order discrimination abilities. This underscores the importance of considering dynamic neural states—shaped by recent experience—when investigating sensory coding principles, as static characterizations of receptive field properties may miss crucial aspects of how neural representations function in natural, non-stationary environments [8,21].

### 4.4. Evolutionary and Comparative Considerations

The demonstration of SSA in the avian Imc adds to growing evidence that fundamental neural computations like adaptation are evolutionarily conserved across vertebrate lineages with independently evolved visual systems [16,21]. Birds and mammals diverged approximately 300 million years ago and have developed substantially different brain architectures—with birds organizing pallial circuits into nuclear clusters rather than the layered cortices of mammals [20,21]. Despite these structural differences, both lineages appear to have converged on similar adaptive mechanisms for optimizing sensory processing, suggesting strong selective pressures for efficient information handling in dynamic environments [5,21,45].

The sensitivity of gamma-band oscillations to adaptation in the avian Imc parallels findings in the mammalian neocortex, where gamma power is modulated by attention, expectation, and stimulus repetition [9,16,29,30]. This convergence may indicate that gamma oscillations represent an evolutionarily optimal solution for coordinating neuronal populations during salient event processing, with similar adaptive principles governing their regulation across distant vertebrate lineages [12,28]. Alternatively, given the independent evolution of avian and mammalian brains, these similarities might represent different implementations of fundamentally similar computational principles—an example of convergent evolution at the neural algorithmic level [16,21]. Further comparative studies, such as those analyzing species-specific adaptation in specific neuronal circuits like the medial nucleus of the trapezoid body, contribute to understanding these conserved mechanisms [46].

## 5. Conclusions

In summary, this study establishes the existence of stimulus-specific adaptation in the avian nucleus Imc, revealing its differential manifestations in spike-rate and LFP signals and its profound impact on object decoding performance. Our findings support a model in which gamma oscillations in the Imc primarily reflect stimulus salience and are highly sensitive to adaptation, while spike timing concurrently encodes both stimulus identity and salience. The dramatic reduction in decoding accuracy following adaptation demonstrates how recent experience dynamically reshapes sensory representations, potentially prioritizing novel stimuli for attention and further processing. These results advance our understanding of evolutionarily conserved adaptive mechanisms in sensory systems and provide new insights into how the avian brain efficiently processes visual information in dynamic environments, providing important support for the search for robust features through the integration of multi-regional and multi-scale analysis methods to accomplish relevant target decoding tasks.

On the other hand, although the constant-order paradigm can reveal differential responses to repeated versus novel stimuli, it cannot fully rule out the influence of non-specific adaptation, fatigue, or drift. Strictly speaking, establishing SSA would still require the adoption of an oddball paradigm with appropriate probability controls. The evidence provided by this study should therefore be regarded as preliminary but important support for visual SSA in the Imc, and future studies employing more rigorous paradigms are needed for further validation.

## Data Availability

The datasets analyzed in the current study are available from the corresponding author on reasonable request.
